# Mental Health Perceptions and Practices of a Cree Community in Northern Ontario: A Qualitative Study

**DOI:** 10.1007/s11469-017-9791-6

**Published:** 2017-07-17

**Authors:** David Danto, Russ Walsh

**Affiliations:** 1 University of Guelph-Humber, Toronto, Ontario Canada; 2 Duquesne University, Pittsburgh, Pennsylvania USA

**Keywords:** Aboriginal, Indigenous, Medicine wheel, Mental health, Psychology, Qualitative, Traditional healing

## Abstract

This project is a qualitative study of the mental health perceptions and practices of one Aboriginal community in the northern Ontario James and Hudson Bay region. Despite a shared history of trauma and oppression with the other five Cree communities in this area, as well as an added trauma of natural disaster and subsequent relocation, this community has been reported to have markedly lower rates of mental health services utilization and suicide. Interviews with eight community leaders and mental health services providers were conducted and analyzed in order to identify the features that distinguish this community. In line with recent recommendations for culturally sensitive and community-compatible research methods, participants’ narratives were organized in terms of the “medicine wheel” of traditional healing. Results showed strong connection to the land and traditions, openness to both traditional and Christian spirituality, community engagement, and shared parenting as strengths valued by a majority of participants.

According to the National Collaborating Centre for Aboriginal Health (2013), the term “Aboriginal” is an inclusive term as used in Canada today and includes First Nations, Inuit, and Metis peoples. The Aboriginal peoples of Canada are those peoples who are indigenous and are the original inhabitants of Canada. In northern Ontario, surrounding James and Hudson Bay, lie six remote Aboriginal communities. These communities range in size from roughly several hundred members to several thousands. The communities have no road access linking them to other communities in the region, and with varying degrees of ease, they can be reached by rail, air, boat, or winter ice road. Within Ontario, the communities along the James and Hudson Bay coast struggle with a host of mental health issues including high rates of suicide, substance abuse, and depression. Amidst these communities, one stands out by virtue of its low rates of suicide and mental health service utilization. This is noteworthy, because this community shares a history of traumatic experiences with its sister communities, but endured in addition the relatively recent trauma of a natural disaster. How is it that one community, despite a similar history of oppression, victimization, and suffering as well as a unique natural disaster, has produced what appear to be more positive mental health outcomes?

In most of the James and Hudson Bay Aboriginal communities, considerable efforts of mental health workers and the allocation of substantial federal and provincial financial resources have had an insufficient impact on persistently high rates of mental illness and mental health problems. According to the Health Status Profile and Environmental Scan (Recollet et al., 2010), suicides and overdoses among teens and those in their twenties in the James and Hudson Bay communities are high with overdoses accounting for 17% of medevacs. The mortality rate in northern Ontario is 18% higher relative to Ontario as a whole. This intransigence has been attributed to the history of forced relocation and confinement, separation of families, oppressive educational practices and sexual abuse, as well as ongoing familial patterns of mental illness.

With respect to mental health services specifically, it has been argued that the discourse and ideals of conventional mental health disciplines are fundamentally incompatible with those of Aboriginal peoples, and hence, efforts to help (via both therapeutic services and relevant research) contribute to further alienation and subjugation of Aboriginal communities (Kirmayer et al. 2000; McCormick 2009). In order to best understand this northern Ontario community with apparent resilience to colonial influence, a suitable research method, more compatible with Aboriginal world views is required.

Generally, the mental health challenges of the James and Hudson Bay communities are well-documented. The Health Status Profile and Environmental Scan: Aboriginal First Nation and Metis (Recollet et al. 2010), which articulates the unique context of northern Ontario communities and identifies multiple challenges within the diverse populations, finds among other conclusions: “…a need for strengthened and coordinated community mental health programs; and, need for regional mental health and acute detoxification inpatient beds in the Weeneebayko area to stabilize and treat mental health and substance abuse patients” (p. 23). As noted by Kirmayer et al. (2000), however, we can expect “wide variations in the levels of mental health and illness in different nations and communities” (p. 36).

Aboriginal youth suicide rates vary dramatically within neighboring communities (Hallett et al. 2007). Kirmayer et al. (2000) examined psychological distress among James Bay Cree communities in Quebec and discovered the suicide rate was no higher than within the general population (The Report of the Advisory Group on Suicide Prevention 2013, p. 26). Indeed, this is not the case in Ontario, where according to community consultations, suicide was the top-ranked priority for the James and Hudson Bay Area (Recollet et al. 2010). However, Hallett et al. (2007) report “youth suicide rates effectively dropped to zero in those few communities in which at least half the band members reported a conversational knowledge of their own “Native language” (p. 393). These findings are further supported by Kirmayer et al. (2003) who evidence “…strengthening ethnocultural identity, community integration and political empowerment can contribute to improving mental health in this population” (S16).

Mental health challenges appear to fluctuate by region and context (Chandler and Lalonde 1998). Understanding these variations and tailoring mental health services accordingly may require idiographically focused research methods. Kirmayer et al. (2009) underscore the need for culturally sensitive and appropriate research regarding mental health. They advocate for “…careful qualitative ethnographic work in order to understand local models of illness and idioms of distress” (p. 15). Chandler and Lalonde (2009) observed that global statistics relating to provincial averages of suicide have been called “actuarial fiction” (p. 221), because they fail to capture the variance in rates by community. In one study, they discovered that “90% of suicides occurred in less than 10% of the bands” (p. 232). Therefore, global actuarial data is insufficient to address the needs of Aboriginal populations.

According to McIvor et al. (2009), Aboriginal communities have asserted “…that their language and culture is at the heart of what makes them unique and what has kept them alive in the face of more than 150 years of colonial rule” (p. 6). Traditionally, Aboriginal communities value the narrative and convey their histories and experiences orally. Therefore, verbal discourse is respected and appreciated within these communities (Kirmayer et al. 2011; McIvor et al. 2009). These values seem most compatible with qualitative research methods grounded in the words and world views of the individuals studied.

A qualitative, idiographic approach stands in contrast to methodologies employing operational definitions, quantification, and broad generalizations that may fit poorly with the world views of participants. As noted by Waldram (2009), “the demands of a predominantly quantitative approach to mental health research have invariably resulted in the reduction of culture to the status of a variable, a methodological development largely rejected by anthropology and one that, ironically, has served to lead us not closer to but further away from an understanding of the role of culture in Aboriginal mental health” (p. 56). To best understand Aboriginal mental health in context, the methodology should be appropriate to the population examined as well as accessible to community members.

There are similarities between communities in their response to colonial oppression among Indigenous populations throughout the world. At a finer level of specificity, however, pressures and injustices have varied and continue to vary by type and degree across all communities. As noted by Kirmayer et al. (2000), “Ongoing transformations of identity and community have led some groups to do well, while others face catastrophe. In many cases, the health of the community appears to be linked to its sense of local control and cultural continuity” (p. 614). Accordingly, Wesley-Esquimaux and Smolewski (2004) have argued for the importance of a community-based approach to addressing these colonial pressures. In order to address the historic trauma which Aboriginal communities have experienced, “…a people-centered and a people-directed approach has to be adopted. The first step to initiate a meaningful healing process is to identify a focal problem that lies at the bottom of contemporary social difficulties in Aboriginal communities” (p. 77). It is this local and people-centered focus for which qualitative and idiographic research seems ideally suited.

Perhaps owing to a tendency in Psychology to follow the Western medical model, many studies on the subject of Aboriginal mental health have focused on pathology or epidemiology (Kirmayer et al. 2009). Information collected from these studies has made an important contribution to the literature by revealing discrepancies between pathology occurrence in Aboriginal populations and the general population (http://www.hc-sc.gc.ca/fniah-spnia/promotion/mental/index-eng.php). However, focusing on pathology carries with it the risk of missing the strengths of many communities who have demonstrated strength in the face of adversity (Kirmayer et al. 2011).

As a result, more recent studies on the subject of Aboriginal mental health have focused on strength and resilience-based research. Research has explored resilience as a fundamental attribute “distinguishing ‘high risk’ individuals who manage to avoid negative outcomes from those who do not” (Stout and Kipling 2003, p. 5). Resilience refers to the interplay between risk factors and protective factors (Fraser and Richman 2001) and between the health of the individual and the health of the community, such that community members are able to withstand the deleterious effects of the dominant culture both historically and currently. There are of course potential shortcomings to the concept of resilience; as highlighted by Luthar et al. (2000), when painted with too broad a brush resilience ignores the considerable variability across individuals, as well as within individuals across time, in facing adversity. Nevertheless, attention to resilience seems an important counterpoint to a predominant focus on negative effects of adverse circumstances, particularly those endured by Aboriginal peoples.

Despite this, the dominant research paradigm in Canada remains natural science-based, quantitative method research. Moreover, reports of community leaders indicate that no research to date has focused on this particular northern Ontario community despite its positive mental health outcomes. This study addresses this lack by employing a hermeneutic qualitative method (Danto 2004; Walsh 1995a, b, 1999) focusing on the community strengths with regard to mental health. A qualitative method elicits narrative accounts from participants so that the nuances of lived experiences are explored in the words and from the perspectives of those participants. A hermeneutic approach seeks to understand those accounts through the horizon of meanings unique to particular communities and contexts.

The authors solicited narratives from community leaders and resident mental health services providers regarding the perceived strengths of their community with respect to mental health, via a culturally sensitive method that privileged the discourse of participants. Nevertheless, a challenge for this study, as for the bulk of research focused on Aboriginal communities, was the “outsider” status of the researchers themselves. That is, despite their interest in and concern for the well-being of Aboriginal communities, they remain unavoidably non-native academically situated Western psychologists. While an argument could be made that this outsider status increases objectivity (an argument implicit in much research focused on Aboriginal communities), in practice, this “from the outside in” orientation runs the greater risk of further oppression and colonization in the name of scientific truth. Qualitative methodology, despite its orientation toward the experiences of participants in their own words, still undertakes the task of organizing and interpreting participants’ accounts, and hence also entails the risk of colonizing participants’ experiences. In order to minimize this risk, the current authors decided to organize and interpret participant narratives in terms of the “medicine wheel” of traditional healing. McCormick (2009) provides the following overview of the medicine wheel:

The Aboriginal medicine wheel is perhaps the best representation of an Aboriginal world-view related to healing. The medicine wheel describes the separate dimensions of the self– mental, physical, emotional, and spiritual – as equal and as parts of a larger whole. The medicine wheel represents the balance that exists between all things. Traditional Aboriginal healing incorporates the physical, social, psychological, and spiritual being. (P. 338)

The medicine wheel identifies as central themes: physical health, intellectual health, spiritual health, and emotional health. According to McCabe (2008), Aboriginal Canadian healing traditionalists view the person as being comprised of these integrated categories and an individual’s health and wellness result when these realms are balanced and integrated. By organizing their qualitative analysis along these lines, the investigators sought to frame their results in culturally appropriate terms, and thereby set the stage for community conversations regarding mental health.

## Method

### Participants

Through his work in northern Ontario, the first author has contact with mental health workers in the region. Over several months, both authors had a series of conversations with these mental health workers regarding their interest in community-oriented and strength-based research that could inform their interventions in these areas. Through these conversations, one community was repeatedly mentioned as one wherein mental health problems seemed less severe despite considerable hardship. Neither author had ever visited this community previously. The band leadership was initially contacted by one of the mental health workers in the area. Subsequently, the first author proposed the research to the community leaders and was invited to carry out the research in this small northern fly-in only community.

A letter from the community’s leadership supporting the proposed study was provided to the first author’s Institutional Research Ethics Board (REB). The authors submitted the proposed study for a full review, and the REB approved the study. The community leadership’s written support was further confirmed orally at the time of data collection, and the study was then carried out in compliance with the First Nations Information Governance Centre OCAP principles of ownership, control, access, and possession.

Eight Aboriginal community members including elders, mental health workers, and other community leaders were recruited via snowball sampling. That is, during an initial meeting with the community’s chief regarding the purpose of this research, several individuals were identified as having perspectives and opinions relevant to the researchers’ questions. At subsequent meetings with these individuals, other relevant persons were identified, and they were then also invited to participate in this research (it is perhaps worth noting that this sampling process resulted in the participation of a prior chief as well as several individuals with perspectives quite distinct from those of the current band leader). To protect the anonymity of participants in this small community, demographic information will not be provided. Recruitment and interviewing took place during a week in August 2012, while the two authors were guests in the community.

### Procedures

The authors arrived via the twice-weekly airplane flight that both delivers needed goods and transports individuals between communities in the area. After obtaining transportation to the band office, they met with the chief, conducted their interview with him, and were given names of several individuals deemed appropriate to reply to the research questions. The authors were then generously provided accommodation at the living quarters typically used by visiting school teachers. This residence served as the location for many of the interviews.

The procedures for this study followed from the authors’ phenomenological and hermeneutic approach to qualitative research (Danto 2004; Walsh 1995a, b, 1999). In practical terms, this approach entails granting primacy to the experience and first-person accounts of participants, and attempting to summarize and interpret those accounts with respect for the contexts of meaning in which they have been lived and understood. For this study, this meant recognizing that (a) for this community, understandings of health and wellness were informed by the medicine wheel, and (b) the authors, as outsiders, would have at best a limited perspective on participants’ experiences, and (c) fidelity to participants’ accounts and humility regarding the researchers’ interpretations would be of crucial importance.

The two researchers met with each of the participants for approximately 90-minute interviews. These semi-structured interviews asked participants to describe the perceived strengths of their community with respect to mental health. The structured questions posed were as follows: (1) “Although your community has faced many hardships, (it) stands out for the health of its members. How does the community facilitate the health and healing of its members?” (2) What do members do to facilitate their own health and healing?” (3) “What are some of the obstacles, past and present to the health and healing of your community?” (4) How does your community overcome these obstacles?” (5) “What are other important features of your community’s health and healing?” While one interviewer posed these initial questions, the second interviewer observed and documented nonverbal aspects of the interview as well as posed follow-up questions (e.g., “Can you tell me more about that?”). All interviews were recorded and transcribed.

Transcribed interviews were then subjected to the following stages of qualitative analysis. The principle investigator, with the help of student research assistants, highlighted keywords and phrases pertaining to the following categories: (1) referents to physical health, (2) referents to intellectual health, (3) referents to spiritual health, (4) referents to emotional health, and (5) referents to aspects not readily categorized within the preceding four categories. These initial categorizations were reviewed by a minimum of two raters, and refinements were made until inter-rater consensus was obtained.

These categorized data were then passed to the second author, who continued the analysis by identifying thematic clusters within each of the five categories. The resulting themes were then reviewed by the first author, who identified apparent inconsistencies, omissions, or alternative interpretations. These points of divergence were then discussed by the first and second author, and themes were refined until inter-researcher consensus was obtained.

## Results

The comments of the eight community members, organized according to the “medicine wheel” of traditional healing, are presented below. The four categorical areas (physical health, intellectual health, spiritual health, and emotional health) were chosen based on the authors’ initial conversations with community mental health workers. In those conversations, the medicine wheel was repeatedly mentioned as a meaningful world view through which community members organized and interpreted their experiences. Hence, in accord with the authors’ hermeneutic approach to this project, analyses were structured along these lines. At the same time, a fifth category of other referents was used to allow for comments and experiences seemingly outside of this structure.

Thematic analyses occurred within the five categories. In other words, participants’ remarks, once sorted by the five categories, were organized according to common themes. Hence, for example, within the category of spiritual health, the authors found recurrent references to participants’ acceptance of religious differences as well as to the value of ceremonies. What follows is a summary of such themes within the broader categories.

### Physical Health

All but one participant explicitly mentioned the surrounding land and their relationship to it as a major strength of the community. Participants saw the land as a mode of access to tradition and cultural practices as well as to healthy food. The participants spoke of hunting and harvesting as providing physical exercise (“when you go in the bush you’re forced to physically work”). They also affirmed the importance of a diet comprised of “healthy” natural food that is hunted and harvested traditionally to benefit from the entirety of the animal (“when people just harvest this for the sake of the meat and throw away a lot of stuff… they’ve lost their culture totally”). They saw disconnection from this diet and way of life as contributing to obesity and diabetes as well as other health concerns. In order to combat the health risks of primarily store-bought meals, inactivity, and disconnection, this community sponsored events and initiatives to keep their children active and connected to their relationship with their natural surroundings.

Participants also spoke of the benefits of physical isolation from colonial culture, in that they had “unchallenged access” to land and resources (“that’s what freedom looks like for us”; “you can walk forever and ever and know that you don’t walk in someone’s yard or property”), could experience their connection to nature (“seeing animals” regularly; “the land is there, it’s open, it’s free – people can feel comfortable where they are”), and encountered minimal imposition of governmental policies. Along these lines, the community did not “administer social assistance” that “makes people totally quit from living off the land.”

### Spiritual Health

All participants identified as a strength the community’s openness to different modes of spiritual practice (“traditional healing and the Catholic community: I think the community has come a long way to accept that it’s all about one God; it’s all about acceptance”; “we still have our elders that go to church”; “our elders, even though they go to church, they’ll come to our ceremonies and participate”). The theme of Christianity, traditional spiritual beliefs, and nonspiritual world views, being able to coexist was an apparent theme, as indicated by the examples above, in addition to the following:

“There is only one religion here. And I think that’s what makes the community strong there’s no division; there’s no squabble between religions.”

“Those are the things that made our people strong: ceremonies. Even when they go to pray in church, and they’re happy, you know? They believe in something higher than them.”

Participants spoke of the importance of ceremonies (“we have traditional ceremonies, and it’s back in our communities”; “those are the things that made our people strong: ceremonies”), community, and their relationship to the land (“This Cree or this person comes from the land and the spirit, the spirit of the land – that’s what makes everybody strong”; “how to live off the land, how to respect people, how to respect everything and everyone”) as sources of comfort (“when you’re right down in despair… when you turn to Christianity and prayer or when you turn to the traditional spiritual stuff it gives you hope again”; “any place is sacred, any place is a good place to pray”), and connection (“I decided to entertain what’s talking directly to me out there … directly connected to the land”).

The land was also identified as a source of spiritual renewal and healing (“there are places where… our elders used to gather… that we draw to, that’s the good stuff we need”), and connection to a “way of life” that goes “back to”, or is still “in touch with”, the “natural flow” and “rhythm of wildlife.” A majority of participants characterized their relationship to the land in spiritual terms (e.g., “the people, the land, it’s like a religion”).

The historical context of these attitudes and practices was also affirmed:

“It was always through ceremonies and people talking to each other – through fire to fire, from shake tent to shake tent, or lodge to lodge. Where are the animals? Where are they today? Where do we need to go? Everybody would migrate as a whole, come from different places to get that and go back. You see… that was our form of communication and life. And we used ceremonies to do that.”

“It’s not just something to talk about. It’s a way of life, you know…”

### Mental Health

Participants spoke of the importance of community cohesion and support for one another, the importance of learning the Cree language, facilitating cultural identity for oneself and children, the importance of family and good parenting, the land in relation to mental health, and traditional healing and programs addressing the residential school system in relation to mental health.

Regarding community cohesion and support in relation to mental health, participants stated the following:

“people help each other out, talk to each other and support each other”

“whenever there’s a crisis, we want to be there for each other… to support one another”

“we truly built a community for ourselves”

“something happens with another family, we help them out; we support people”

“everybody, even if they disagree… when it comes to a crisis and someone needs help… that’s where your strength is: the whole community comes together”

“everyone still watches out for everyone”

“I focus on the positive side, like building good relationships”

Participants also spoke of the importance of maintaining the Cree language, although they also acknowledged the challenges in doing so now that there is not Cree instruction in the school.

In one way or another, all participants affirmed the importance of their cultural identity for themselves and/or the children of the community. More generally, participants affirmed the importance of “not forgetting who we are”, and having a “sense of identity” or “sense of pride” for the “traditional way of life”:

“We have a belief. Like I’m not going to give it a word of religion or culture. No, it’s a way of life, you know. It always was in the beginning, and it is today.”

“We still understand where we come from”

“(The Elders) try to teach the young people how it was done back then”

“The right question is, how did you come about to be, to be the way we are today?”

“To have… good citizenship you need a … solid cultural background… to include cultural, ancestral history”

“It’s not of arrogance, or ignorance either; it’s just proud of who we are”

Participants also identified the sense of family, “good parenting”, and community concern for the youth as key aspects of mental health:

“the family roots are so powerful here, it’s unbelievable”

“we don’t run after social programming… instead, we talk to each other as parents”

“parental authority – not so much authority but responsibility over children… good control over your family”

“kids are wise, and need direction by adults”

“our youth has a sense of identity; we support them, and they have pride”

“training (the young people) to be proper adults… to have early responsibilities…these include different character building for girls and boys… respect for women, respect for men, that kind of thing”

“parents they just don’t like their youth walking around at night; if someone’s late, they’ll walk around and look for them”

“you keep talking to your children and showing them, because in our culture we learn through observing things”

“and we watch out for our children: like, if I see her child doing something he wasn’t supposed to do, you say, ‘hey, you can’t do that’”

“Spend(ing) time out on the land” as a way of caring for one’s mental health emerged as a theme:

“you have to come home; get rooted and go back out again”

“go out in the bush, refresh my memories; when I come back everything is clear”

“to know the land… you know you’re capable of things other kids aren’t; knowing where I came from, what I’m capable of”

Regarding community “healing and treatment programs”, participants spoke of “traditional healing”, including “monthly visits from traditional healers” and “traditional ceremonies”, as well as “sharing circles” and other mental health interventions by “crisis coordinators” and “front-line workers”. They also described an “internal network of (support) dealing with the trauma of residential schools.” More broadly, participants described community programs for promoting healthy lifestyles, particularly among the young people in the community.

### Emotional Health

Participants conveyed an optimistic resilience and spoke of hopefulness, which entails “focusing on the future” and the ability to “accept whatever comes” and recognize “the natural cycle of things” in order to “build a healthy community for the future generation”, participants stated, “we leave the bad behind and we transpire and hope for the future”, and “the strength, I guess, would be hope, the hope for tomorrow.” Participants also conveyed that community members are happy owing to their spiritual connectedness (“they’re happy, you know? They believe in something higher than them”).

“back to the land: when you’re there, it’s like your spirit, your mind, and your physical well-being – everything improves when you’re out there; it’s like you rejuvenate while you’re out there”

### “Other” Participant Responses

Participants offered several comments not readily organized according to the quadrants of the medicine wheel. Most notable among these was an elder’s characterization of “submission” as a community strength. His comments in this regard underscored openness toward and acceptance of whatever comes, particularly that resulting from natural fluctuations in weather and the availability of food. In this sense, submission could be equated with a sense of rhythm or synchrony with the flow of nature. However, the elder also spoke of how “whatever comes” in recent history entailed the imposition of Western ways and values, and that submission in this regard posed opportunities for integration of new practices (for example, Christian spirituality) as well as the potential for subjugation and alienation from tradition. What was most striking was the elder’s acceptance of both the light and dark sides of submission in a way that portrayed them as inextricable from one another.

Other participants spoke of challenges faced by the community, such as the changing lifestyles of young people (including alcohol consumption), the imposition of the federal government (particularly in the form of “one size fits all” programs), and as previously indicated a lack of Cree language education. While these were always presented in the context of community strength to face these challenges, they were nonetheless significant areas of concern for some participants.

## Discussion

The authors view the findings of this study as consistent with prior research carried out in Canada regarding factors that promote resilience and mental health in Aboriginal communities. Participant responses emphasized cultural continuity factors and an overall sense of ethnocultural identity. The present study, while admittedly conducted by researchers outside of the community, sought to ground its findings in the words of community members and the cultural framework of the medicine wheel. This posed many challenges, as the categorization of participants’ remarks into specific dimensions of the medicine wheel was rarely straightforward. This was to be expected; however, as the medicine wheel represents a holistic and multi-dimensional perspective, with phenomena potentially having great relevance to multiple categories.

In the instances where participant remarks seemed to bridge multiple categories, the same phrases were categorized multiple times (i.e., included in more than one category). The importance of the land for example appeared relevant across all medicine wheel categories. Nonetheless, these methodological choice points underscore the inevitable challenges of studying Aboriginal perspectives from positions outside of their community and culture. The band chief stated, “our lifestyle is a circle”, and contrasted this with an orientation to “stay in one square”. The task of categorizing, even in accord with a culturally accepted framework, still runs the risk of imposing “squares” on “circular” experiences.

This imposition of a non-native structure on the more fluid world views of Aboriginal peoples is an almost indelible part of any research. While qualitative methods provide some important safeguards, particularly with respect to the imposition of abstract and numerical generalizations, researchers employing this paradigm must be equally vigilant regarding their potential for colonizing conclusions. Researchers can never forget that as outsiders, they must tread lightly.

The limitations of this study are of course many. The authors interviewed a small number of people, in one community, during a snapshot in time, and despite their hermeneutic approach were and remain clearly outsiders. The authors recall vividly the greeting of many community members encountered, who very pleasantly but pointedly asked, “what are you doing here?” Hopefully, what the authors did there was to contribute in some small way to ways of thinking about and studying Aboriginal mental health. The authors do not view these findings as necessarily generalizable truths, but rather as the start of a conversation; a conversation which, to be meaningful and helpful, must continue to include the community members themselves.

To the authors’ eyes, the most notable finding in this study was the ways in which connection to the land was interwoven throughout all aspects of the medicine wheel. Participants’ comments regarding physical, spiritual, mental, and emotional health often referred to attitudes and practices that affirmed a fundamental connection to their land. This connection informed individual and community efforts to maintain well-being (such as regular hunting trips and the acculturation of young people in this regard), and also seemed to provide a bridge between different spiritual beliefs (that is, both Christians and traditionalists saw the land as foundational to their faith). Related to this was the almost universal concern for “challenges” that entailed some form of disconnect from the land (e.g., relying on store-bought food rather than hunted food, as well as the increasing role of television and video gaming in the lives of young people). This may hold implications for health and healing initiatives both within and beyond this community. If a sense of connection to the land is a central feature of well-being, then it may need to be a central feature of mental health interventions.

Another strength affirmed by all participants was the community’s acceptance of differences among members regarding spiritual practices. Considering the potential for religious conflict based in contrasting beliefs (which at their most extreme could characterize one group as pagan, or the other as abandoning spiritual tradition) as well as the history of trauma resulting from residential schools, this finding suggests a high level of community cohesion that allows for differences within that community. This cohesion was explicitly mentioned by the vast majority of participants and was also apparent in comments regarding strong family relationships and shared culture. Indeed, it is possible to see a common thread of community cohesion woven throughout the vast majority of participants’ remarks. This cohesion was evident in community activities and programs, as well as in acknowledgement of shared culture and history. It may well be the case that members’ shared connection to the land and ready access to the land was sufficiently strong to tolerate differences that might otherwise polarize a community. And from this, shared sense of connection may follow the sense of hope expressed by most of the current participants.

Two other factors may also play a role in this particular community’s strong sense of identity. First is the relative isolation of this community, such that “outside influence” is moderated in favor of greater local identity and control. Second is the rather recent shared trauma of natural disaster and relocation, which required a pulling together of community resources in a way that more diffuse challenges and traumas may not. These two factors may ameliorate the potential for cultural discontinuity, which has been shown to be a precursor to high rates of depression, alcoholism, suicide, and violence and related to intergenerational loss and grief (Tousignant and Sioui 2009; Chandler and Lalonde 2009). As noted by Kirmayer, Brass, and Valaskakis (2009), “collective historical grief has been used to acknowledge the profound sense of loss of continuity and tradition as a people” (p. 465). A sense of sharing this grief, both in terms voicing it to one another and experiencing it as common across community members, may be an important part of healing and affirming one’s sense of community.

To the degree that these factors are foundational to the strengths of this community, there may be implications for more general intervention and prevention programs. Specifically, these findings suggest that when communities can unite to face a set of problems and have a fair degree of autonomy (i.e., freedom from outsider influence) in responding to those problems, they may be best able to recognize and draw upon their shared resilience and communal spirit. For those wishing to facilitate this resilience and spirit, the challenge is to do so in a way that affirms rather than usurps the community’s independence.

The results of this study show the potential value of qualitative, idiographic research exploring the strength and resilience of particular Aboriginal communities. By privileging community members’ perspectives in their own words, this research affirms the value of community-centered approaches that build research and intervention programs from the “inside out”. Many of the strengths identified by participants, such as hopefulness, community engagement, and shared parenting, could be perceived quite differently if advocated by agencies and programs outside of the community. That is, interventions promoting hope and/or shared parenting “from the outside in” are likely to be perceived as naïve, oppressive, and colonizing. In order to minimize this risk, qualitative research seems better suited to the goal of developing culturally sensitive and culturally rooted health and healing initiatives. But researchers must nonetheless remain aware of these inherent risks and as allies or advocates try to step lightly.
